# Wheat Intercropping Enhances the Resistance of Watermelon to *Fusarium* Wilt

**DOI:** 10.3389/fpls.2018.00696

**Published:** 2018-05-25

**Authors:** Huifang Lv, Haishun Cao, Muhammad A. Nawaz, Hamza Sohail, Yuan Huang, Fei Cheng, Qiusheng Kong, Zhilong Bie

**Affiliations:** ^1^Key Laboratory of Horticultural Plant Biology, Ministry of Education, College of Horticulture and Forestry Sciences, Huazhong Agricultural University, Wuhan, China; ^2^School of Fine Arts and Design, Chizhou University, Chizhou, China

**Keywords:** intercropping, root exudates, disease resistance, coumaric acid, *Fusarium* Wilt, *Fusarium oxysporum* f. sp. *Niveum*, watermelon, wheat

## Abstract

A fungus *Fusarium oxysporum* F. sp. *niveum* (FON) is the causal organism of *Fusarium* wilt in watermelon. In this study, we evaluated the effect of wheat intercropping on the *Fusarium* wilt of watermelon. Our results showed that wheat intercropping decreases the incidence of *Fusarium* wilt of watermelon, likely due to the secretion of coumaric acid from the roots of wheat that dramatically inhibits FON spore germination, sporulation, and growth. The secretion of *p*-hydroxybenzoic acid, ferulic acid, and cinnamic acid from the roots of watermelon stimulates FON spore germination, sporulation, and growth. The secretion of phenolic acids and organic acids from the roots of watermelon is also promoted by FON infection. However, secretion of phenolic acids and organic acids from the roots of watermelon is substantially reduced under wheat intercropping systems. FON infection increases the accumulation of free and conjugated salicylic acid (SA) in watermelon grown under wheat intercropping systems through isochorismate (ICS) and phenylalanine ammonia-lyase (PAL) pathways. Furthermore, wheat intercropping up-regulates the expression of disease-and defense-responsive genes and improves the activities of corresponding pathogenesis-related (PR) enzymes in the roots of watermelon. In conclusion, the secretion of coumaric acid from the roots of wheat and changes in the composition of phenolic acid and organic acid secretion from the roots of watermelon suppress *Fusarium* wilt of watermelon under wheat intercropping system. Meanwhile, wheat intercropping also enhanced the resistance of watermelon to FON by up-regulating the expression of disease-and defense-responsive genes in watermelon.

## Introduction

Watermelon (*Citrullus lanatus* L.) is an important crop that is widely cultivated across the world. Watermelon plants are sensitive to wilt disease caused by *Fusarium oxysporum* f. sp. *Niveum* (FON), which poses a serious threat to watermelon cultivation. Different techniques, such as grafting onto disease-resistant rootstocks (Ling et al., 2013; Bie et al., 2017; Nawaz et al., 2017), biological control (Zhang et al., 2011; Zhao et al., 2014), and use of disease-resistant cultivars (Bai and Shaner, 1996) are utilized to overcome this menace. Intercropping is used to control different pathogenic problems such as *Fusarium* wilt because this is a safe and efficient method (Ren et al., 2008; Zhang et al., 2013; Gao et al., 2014).

Root exudates play an important role in the communication between soil microbes and plant roots (De-la-Pena et al., 2008). The quantity and composition of root exudates are affected by the plant species, growth conditions, and developmental stage of plants. A small change in the composition and quantity of root exudates causes major changes in the population of microorganisms in the root zone (Badri and Vivanco, 2009; Ling et al., 2011). In plant-soil pathogen feedback, some allelochemicals released by the roots promote the growth of soil-borne pathogens whereas some allelochemicals inhibit the growth of pathogens (Dixon, 2001; D’Auria and Gershenzon, 2005). Phenolic acids released by the roots act as signaling molecules or attractants (Lanoue et al., 2010; Mandal et al., 2010). Phenolic acids, such as *p*-coumaric acid, can inhibit FON colonization (Hao et al., 2010; Zhou and Wu, 2012c). The interaction between plant root exudates and soil-borne pathogens shows that different types and concentrations of phenolic acids in root exudates differ in their mode of action on pathogens such as *F. oxysporum* (Hao et al., 2010; Ling et al., 2013).

Root exudates contain organic acids. Watermelon roots secrete organic acids such as malic acid and citric acid, which promote the colonization of *Paenibacillus polymyxa* SQR-21, a biocontrol-agent with a broad host range (Ling et al., 2011). Similarly, Liu et al. (2014) found that an organic acid, such as fumaric acid, promotes the biofilm formation of *Bacillus amyloliquefaciens* SQR9 under laboratory conditions. *B. amyloliquefaciens* is utilized in the agricultural sector to control different bacterial diseases caused by *Ralstonia solanacearum*, *Pythium*, *Rhizoctonia solani*, *Alternaria tenuissima tenuissima*, and *Fusarium* (Huang et al., 2014; Tan et al., 2015). Oxalic acid is also considered a pathogenic factor for fungal infection in plants (Schoonbeek et al., 2007). According to a report by Ren et al. (2015), the amount of phenolic acids and organic acids secreted from the roots of watermelon is increased when roots are infected by FON. However, information regarding the potential of wheat intercropping to control *Fusarium* wilt of watermelon through the regulation of root exudate is limited. Thus, we hypothesized that wheat intercropping can effectively control the *Fusarium* wilt of watermelon by modifying the phenolic acid and organic acid content secreted by the roots of watermelon.

Salicylic acid (SA) is an important signaling molecule that activates the defense system of a plant under different biotic and abiotic stress conditions. SA is synthesized in plants by two different pathways, namely the isochorismate (ICS) and phenylalanine ammonia-lyase (PAL) pathways. When plants are attacked by a disease, SA acts as an immune signal to induce systemic acquired resistance (SAR). SA also triggers the expression of defense-related genes and induces stress tolerance under biotic stress conditions (Kumar, 2014). Pathogenesis-related genes (*PR* genes) are involved in plant defense. Two important markers, *PR1* and *PR2*, are involved in the SA-mediated defense system (Ward et al., 1991; Van Loon, 1997; Rairdan and Delaney, 2002). *NPR1* is necessary for pathogenesis defense responses (Delaney et al., 1995), and it is considered to be included in a complex with the SA receptor (Fu et al., 2012; Wu et al., 2012). Several studies report that *WRKY* transcription factors play an important role in plant defense against pathogen infection (Eulgem et al., 1999; Ülker and Somssich, 2004; Zhang and Wang, 2005).

In this study, we assessed the effect of wheat intercropping on *Fusarium* wilt of watermelon, and the effect of wheat and watermelon root exudates on the growth of FON. The composition of watermelon and wheat root exudates and the effect of these exudates on the growth of FON were determined. The composition of watermelon root exudates after FON infection was also analyzed. Additionally, we measured the expression of watermelon genes involved in disease resistance after FON infection. The overall objective of this study was to understand the mechanism that how wheat intercropping controls *Fusarium* wilt of watermelon.

## Materials and Methods

### Plant Material and Pathogen Strains

Watermelon [*Citrullus lanatus* (Thunb.) Matsum. and Nakai var. Zaojia 8424] and wheat (*Triticum aestivum* L. var. Emai 18) were used as plant materials. FON was isolated from an infected watermelon plot.

### Greenhouse Experiment

Plastic greenhouse situated at Huazhong Agricultural University (113°41′-115°05′ E, 29°58′-31°22 N) was used for these experiments from March 2015 to June 2015. The experiment consisted of four treatments: (1) watermelon monocropping (M) in which one watermelon seedling was grown alone in the pot; (2) watermelon monocropping with FON inoculation (MF); (3) watermelon/wheat intercropping (I) in which one watermelon seedling was grown with 40 wheat seedlings (the distance between the watermelon and wheat seedlings was 10 cm); and (4) watermelon/wheat intercropping with FON inoculation (IF). A total of 30 pots were utilized in each treatment and all the treatments were replicated three times.

Watermelon seeds were germinated at 30°C for 48 h. After the emergence of true leaves, transplantation of uniform plants was done in plastic pots (34 cm diameter, 24 cm height) containing 8 L of moist inoculated mixture of peat and vermiculite (v/v, 1:1). The Hoagland solution (Hoagland and Arnon, 1950) was used to fulfill the nutritional requirements of the plants. The watermelon plants of the MF and IF groups were inoculated with FON spores (2.0 × 10^6^ mL^-1^; 100 mL per pot) near the roots after 7 days of transplanting.

Watermelon root samples were harvested at different intervals after FON inoculation and immediately frozen in liquid nitrogen and then stored at -80°C. The rhizosphere medium samples were taken by gently mixing the media near the roots. The media containing the samples were stored at -70°C for quantification of the FON population. Roots of watermelon samples were washed with tap water and then dipped in a container with 2 L of deionized water. Plants were placed in a phytotron at 28°C/16°C for 10 h to collect root exudates, which were subsequently filtered by 0.45 μm Millipore membranes and evaporated by rotary evaporator at 40°C before the samples were stored at -20°C. Each treatment was cultured in triplicate.

### Assessment of Disease Incidence

Total plants used in the experiment were divided by diseased plants to get the percentage of disease incidence (Wu et al., 2009).

### Plant Dry Biomass Analysis

To measure the dry weight of whole plants, six plants per replicate were harvested on days 15 and 25 after FON inoculation. The adhering media was removed from the roots with tap water after the collection. The plants were placed for drying in an oven for 15 min at 105°C and then placed at 70°C for 3 days. The dry weight of each plant was measured with an electric balance (MSE24P-1-CE-DA, Cubis, Sortorious, Göttingen, Germany).

### Soil DNA Extraction and qPCR Amplification

The PowerSoil DNA isolation kit was used for the extraction of genomic DNA according to the manufacturer’s instructions (Omega Bio-Tek, Inc., Norcross, GA, United States). Real-time PCR (qRT-PCR) was used for the estimation of FON populations. The specific primers FON-1 and FON-2 were used for the quantification of FON (Zhang et al., 2005). Quantitative PCR (qPCR) amplifications were conducted on a QuantStudio^TM^ 7 Flex Real-Time PCR System using qPCR SuperMix. The reaction mixture was 10 μL containing 5 μL of 2 × Top Green qPCR SuperMix, 10 pM 0.5 μL of each primer, 1 μL of template DNA (100 ng/μL) and 3 μL of sterile water. The PCR program was run for 5 min at 94°C, followed by 40 cycles of 94°C for 30 s, 58°C for 15 s, 72°C for 15 s, and a final elongation at 72°C for 7 min. Plasmid strands for the estimation of FON were generated by cloning the target gene from FON genomic DNA. The standard curves were created by Zhou and Wu (2012a). FON population was expressed as number of copies (Wakelin et al., 2008).

### SA Measurement

The root tissues (0.3 g) were ground in liquid nitrogen and acetate buffer (0.1 M, pH 5.6) was added (2.5 mL/mg tissue), mixed, and centrifuged at 4°C for 20 min at 12 000 rpm. The supernatant was used for the measurement of SA according to the method described by Defraia et al. (2008) and Cheng et al. (2016). The estimation of PAL was done according to the method described by Edwards and Kessmann (1992). The activity of benzoic acid-2-hydroxylase (BA2H) was measured according to the method described by Pan et al. (2006).

### Total RNA Extraction and Gene Expression Analysis

The RNA from watermelon roots was extracted by TransZol reagent (TransGen) Biotech, Inc., according to the manufacturer’s instructions. The extracted RNA was dissolved with diethylpyrocarbonate-treated water. 1 μg of total RNA was synthesized for the cDNA template by using HiScriptII QRT Super Mix for qPCR. qRT-PCR analysis was performed after FON inoculation as described by Cheng et al. (2016). CICAC and CITUA were used as reference genes (internal control) (Kong et al., 2014). The primers were designed according to the method described for the *CIPAL* gene family (Dong and Shang, 2013), and the primers for specific genes of watermelon were designed from the coding DNA sequences (CDSs) (v1) in the Cucurbit Genomics Database^1^ (**Supplementary Table S1**). These primers were utilized for amplification and data was analyzed according to the method described by Livak and Schmittgen (2001).

### Determination of Chitinase and β-1,3-Glucanase Activities

Fink et al. (1988) and Schraudner et al. (1992)methods were utilized for the enzymatic assays. Root tissues (0.3 g) were ground to a powder in liquid nitrogen. The homogenate was centrifuged at 4°C for 20 min at 12000 rpm after the addition of ice cold PBS buffer (50 mM, pH 7.8), 0.2 mM EDTA, 2% (w/v) PVP, and 2 mM AsA.

### Collection of Root Exudates

The seedlings of watermelon and wheat having 5 to 6 leaves were washed with distilled water and shifted into a glass container covered with a black plastic sheet to protect from light and contamination. Plants from different treatments were cultured in triplicate. Plantlets were placed in a phytotron at 28°C/16°C for 48 h to collect root exudates and filtered with 0.45 μm Millipore membranes and evaporated by rotary evaporator at 40°C. Then the samples were stored at -20°C. For producing the concentrated extract (1 g root dry weight mL^-1^) the root exudates were dissolved in deionized water for the bioassay and analysis of organic components (Hao et al., 2010).

### Effect of Root Exudates on FON Spore Germination

The conidial suspension was prepared by transferring a block of stock culture to potato dextrose agar (PDA) medium for 5 days at 28°C under dark conditions. Colonies were grown in Potato Dextrose Broth and incubated in a table-top concentrator at 28°C and 125 rev min^-1^ for 7 days. A cheese cloth with three layers was used for the filtration of the suspension and the final concentration was adjusted to 1 × 10^3^ conidia/mL in sterile deionized water using hemocytometer. Different concentrations of root exudates were added on concave glass slides to check their effect on spore germination. The collected exudates from the roots of watermelon and wheat seedlings were diluted to three concentrations (0.01, 0.05, and 0.1 g mL^-1^) and filtered through 0.22 μm sterile filters. Microscopic analysis was performed after 8 h for the determination of spore germination. The effect of root exudates on FON spore germination was calculated as the relative germination impact index according to the methods described by Ling et al. (2013).

### Effect of Root Exudates on FON Sporulation

To determine pathogen sporulation, Bilay’s medium (Booth, 1971) was utilized. 1 mL of root exudate from watermelon and wheat having different concentrations (0.01, 0.05, and 0.1 g mL^-1^) was used for determination of pathogen sporulation. 9 mL of distilled water was added to bring the volume up to 10 mL per 50 mL Erlenmeyer flask. The spore suspension (100 μL) 2 × 10^5^ spores mL^-1^ was inoculated and incubated in a shaker at 28°C at 150 rpm for 2 days. After the incubation period, the culture (100 μL) was spread onto the PDA. Plates were incubated for 4 days at 28°C. After incubation, the colonies were counted and converted in to number of condia in a liquid culture. The relative sporulation impact index was calculated according to the formula: sporulation impact index = (number of sporulation in a treatment-control)/control.

### Effect of Root Exudates on FON Mycelial Growth

To assess the effect of root exudates of watermelon and wheat on FON mycelial growth, plugs (5 mm) containing fungus were cut from the margins and placed in the center of petri dishes containing 19 mL of PDA mixture. Then the mixture was incubated at 28°C under dark conditions with 1 mL of root exudates of watermelon and wheat at concentrations of 0.01, 0.05, and 0.1 g mL^-1^, and 1 mL sterile deionized water. Diameter of colonies was measured in five different directions from each incubated plate up to 7 days. The relative growth impact index was calculated according to the method described by Ling et al. (2013).

### Analysis of Root Exudates by HPLC

Root exudates were filtered with a 0.22 μm filter before the estimation of phenolic acid and organic acid composition and content. HPLC-2998 system (Waters 1525, United States) was used to identify phenolic acids and organic acids from the root exudates of watermelon and wheat. The phenolic acids were determined with a Welchrom-C18 column (4.0 mm × 250 mm, 5-Micron, Agilent, United States) according to the method described by Hao et al. (2010). The mobile phase consisted of methyl alcohol (A) and 1% acetic acid solution (pH 2.59) (B) with a gradient elution. The wavelength of UV detector was adjusted to 280 nm. The column temperature was kept at 30°C. For HPLC analysis, standards such as cinnamic acid, coumaric acid, *p*-hydroxybenzoic acid, ferulic acid, phthalic acid, SA, and syringic acid were used. Chromatography for standard compounds was checked alone and in mixtures. The major peaks were recorded at different retention times.

The analytical conditions for organic acids were as follows: chromatographic column, Zorbax SB-Aq (4.6 mm × 250 mm, 5 μm); detector wavelength, 215 nm; column temperature, 35°C; flow velocity, 0.5 mL min^-1^; and injection volume, 10 μL. Methyl alcohol (A) and 50 mmol/L KH_2_PO_4_-H_3_PO_4_ buffer solution (pH 2.45) (B) were used as mobile phases with an isocratic elution. Standard organic acids include oxalic acid, malic acid, citric acid, succinic acid, and fumaric acid for HPLC analysis.

### Effect of *p*-Hydroxybenzoic Acid, Ferulic Acid, Cinnamic Acid, and Coumaric Acid on FON Spore Germination, Sporulation, and Growth

The spores were grown on concave glass slides with a series of dilutions of *p*-hydroxybenzoic acid, ferulic acid, cinnamic acid, and coumaric acids for the spore germination test. The sporulation of FON was measured using Bilay’s medium (Booth, 1971) with a series of dilutions of *p*-hydroxybenzoic acid, ferulic acid, cinnamic acid, and coumaric acid for the sporulation test. The plugs of FON agar were cultured on PDA plates containing a series of dilutions of *p-*hydroxybenzoic acid, ferulic acid, cinnamic acid, and coumaric acid for the mycelial growth test. All the acid solutions were filtered (0.22 mm) and added to sterilized substrate. The control was sterilized water without acids. All the concentrations of acids were tested by using three replicates. The germination, sporulation, and growth impact index were also calculated as described earlier.

### Statistical Analysis

Statistical analysis was performed using SPSS statistical software (IBM SPSS 19, IBM Corporation, New York, NY, United States). Differences between two treatments were tested by independent sample *t*-test at *P* < 0.05 (**Tables 1**, **2**). ANOVA, followed by Tukey’s test (*P* < 0.05) was used to analyze the differences among treatment means. A general model fitting function for analysis of dose response data was used to analyze the results of phenolic acids by the drc package of R. All the values were expressed as mean ± standard error.

**Table 1:** Quantitative analysis of phenolic acids in the root exudates of watermelon and wheat plants (μg g^-1^ root DW) by HPLC.

Phenolic acids	Watermelon	Wheat
Coumaric acid	0^b^	5.1 ± 0.5a
*P-*hydroxy benzoic	10.6 ± 0.6^a^	0b
Phthalic acid	4.2 ± 0.3^a^	1.3 ± 0.1b
Syringic acid	0.9 ± 0.0^b^	1.6 ± 0.1a
Ferulic acid	1.1 ± 0.1^a^	0b
Salicylic acid	9.8 ± 0.2^a^	2.9 ± 0.1b
Cinnamic acid	3.1 ± 0.1^a^	0b
Total content	29.7 ± 1.3^a^	10.9 ± 0.8b


**“0” means not detectable. All values are presented as mean ± SE. The different letters on the mean values of the same phenolic acid indicate significant differences between the treatments (P < 0.05, independent sample t-test).**

**Table 2:** Quantitative analysis of organic acids in the root exudates of watermelon and wheat plants (μg g^-1^ root DW) by HPLC.

Organic acids	Watermelon	Wheat
Oxalic acid	1305.1 ± 160.3^a^	414.3 ± 19.0b
Malic acid	8169.7 ± 692.9^a^	579.8 ± 14.1b
Citric acid	3354.7 ± 374.3^a^	32.4 ± 1.5b
Succinic acid	1997.7 ± 160.1^a^	0b
Fumaric acid	13.1 ± 1.4^a^	2.4 ± 0.3b
Total content	14840.3 ± 1389.0^a^	1028.9 ± 34.9b


**“0” means not detectable. All values are presented as mean ± SE. The different letters on the mean values of the same organic acid indicate significant differences between the treatments (P < 0.05, independent sample t-test).**

## Results

### Effect of Wheat Intercropping on Plant Growth and *Fusarium* Wilt of Watermelon

**FIGURE 1:**
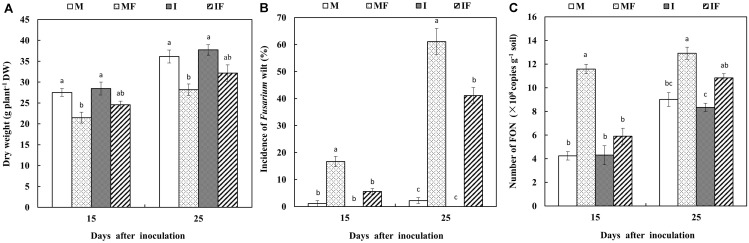
Dry weight of watermelon **(A)**, incidence of *Fusarium* wilt of watermelon **(B)**, and the number of FON in the rhizosphere of watermelon **(C)** on day 15 and day 25 after FON inoculation. M, watermelon monocropping; MF, watermelon monocropping with FON inoculation; I, watermelon/wheat intercropping; IF, watermelon/wheat intercropping with FON inoculation. All values are presented as mean ± SE. The different letters on the mean values of the same day indicate significant differences among the treatments (*P* < 0.05, Tukey’s test).

The FON inoculation reduced the dry weight of watermelon plants. In a monocropping system, slow growth of watermelon plants was observed compared with the intercropping system (**Supplementary Figure S1**). Similarly, the dry weight of watermelon was significantly reduced in the monocropping system by FON inoculation. However, no difference was observed in each cropping system after inoculation with FON (**Figure 1A**).

Wheat-intercropped watermelon plants also showed less disease incidence (5.6 and 41.1%) compared with monocropped watermelon plants (16.7 and 61.1%) on days 15 and 25 after FON inoculation, respectively (**Figure 1B** and **Supplementary Figure S2**). In monocropped watermelon plants the disease symptoms were also observed earlier (on day 7) compared with wheat-intercropped watermelon plants (on day 12) after FON inoculation.

The number of FON DNA copies was higher in monocropping system compared with the intercropping system on days 15 and 25 after FON inoculation. Maximum population of FON was observed in monocropping system (12.9 × 10^8^ copies g^-1^ soil) on day 25 after inoculation with FON (**Figure 1C**).

### Effect of Root Exudates of Watermelon and Wheat on Spore Germination, Sporulation, and Growth of FON

**FIGURE 2:**
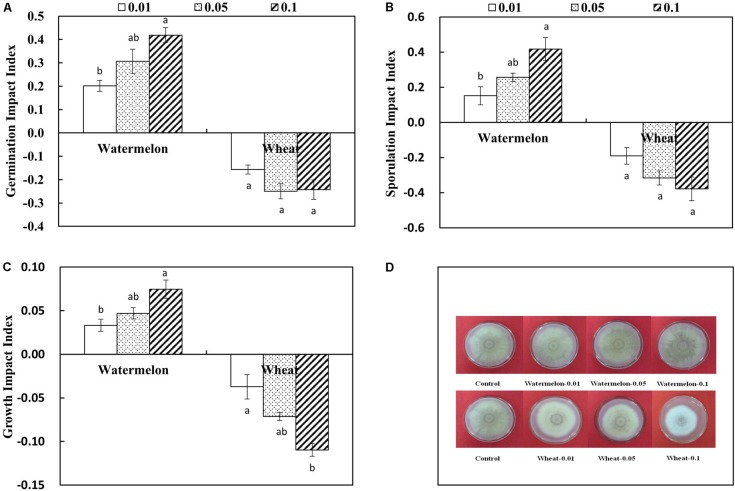
Effect of different concentrations of root exudates collected from the roots of watermelon and wheat on FON germination impact index **(A)**, sporulation impact index **(B)**, growth impact index **(C)**, and growth **(D)**. Germination impact index = (the spore germination number in treatment – control)/control. Sporulation impact index = (the sporulation number in treatment – control)/control. Growth impact index = (the growth diameter in treatment – control)/control. The values (0.01, 0.05, and 0.1) represent the concentrations of root exudates of watermelon and wheat that were 0.01, 0.05, and 0.1 g mL^-1^, respectively. All values are presented as mean ± SE. The different letters on the mean values of the same sample indicate significant differences among treatments (*P* < 0.05, Tukey’s test).

The germination, sporulation, and growth of FON were apparently promoted by watermelon root exudates compared with the control. A stimulatory effect of root exudates was also observed that gradually increased as the concentration increased from 0.01 to 0.1 g mL^-1^. On the contrary, the germination, sporulation, and growth index of FON were apparently inhibited by the addition of wheat root exudates. These inhibitory effects were enhanced with increasing concentration of wheat root exudates (**Figures 2A–D**).

### Composition of Root Exudates of Watermelon and Wheat

The root exudates were collected from watermelon and wheat and analyzed by HPLC (**Supplementary Figures S3**, **S4**). Phthalic acid, SA, and total phenolic acids present in the root exudates of wheat were three times less compared with watermelon; however, syringic acid content of root exudates of wheat was 1.8-fold compared with root exudates of watermelon (**Table 1**). Coumaric acid was only detected in the root exudates of wheat, whereas *p*-hydroxybenzoic acid, ferulic acid, and cinnamic acid were only detected in the root exudates of watermelon (**Supplementary Figures S3B,C**). The concentration of coumaric acid was highest (46.8%) among the phenolic acids identified in the root exudates of wheat (**Table 1**).

According to the retention time of the standards, four kinds of organic acids, namely oxalic acid, malic acid, citric acid, and fumaric acid, were detected in the root exudates of watermelon and wheat (**Supplementary Figures S4B,C**). However, only succinic acid was found in the root exudates of watermelon. The content of different organic acids was higher in the root exudates of watermelon compared with wheat. The concentration of malic acid in particular in the root exudates of watermelon was higher (55.1%) compared with that of other organic acids. The total organic acid content in the root exudates of watermelon (1.5 × 10^4^ μg⋅g^-1^ root DW) was 14-fold higher compared with root exudates of wheat (1.0 × 10^3^ μg⋅g^-1^ root DW) (**Table 2**).

### Effect of *p*-Hydroxybenzoic Acid, Ferulic Acid, Cinnamic Acid, and Coumaric Acid on Spore Germination, Sporulation, and Growth of FON

**FIGURE 3:**
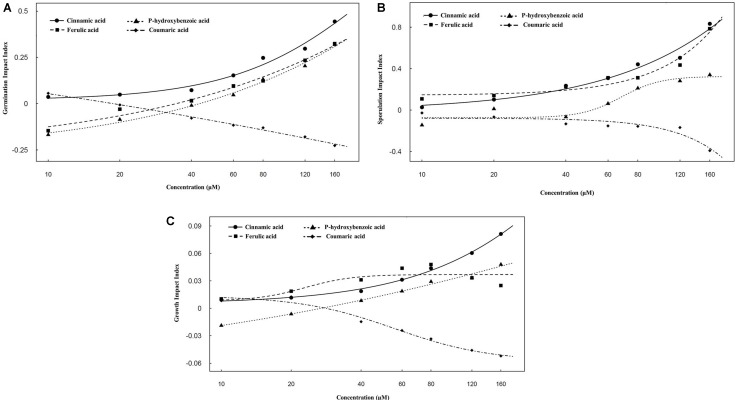
Effect of different concentrations of *p*-hydroxybenzoic acid, ferulic acid, cinnamic acid, and coumaric acid on FON germination impact index **(A)**, sporulation impact index **(B)**, and growth impact index **(C)**. Germination impact index = (the spore germination number in treatment – control)/control. Sporulation impact index = (the sporulation number in treatment – control)/control. Growth impact index = (the growth diameter in treatment – control)/control. Values of 10, 20, 40, 60, 80, 120, and 160 represent the concentrations of *p*-hydroxybenzoic acid, ferulic acid, cinnamic acid, and coumaric acid that were 10, 20, 40, 60, 80, 120, and 160 μM, respectively. All values are presented as mean ± SE.

We hypothesized that these acids might play a role in disease incidence of FON. Therefore, the effect of *p*-hydroxybenzoic acid, ferulic acid, cinnamic acid, and coumaric acid on FON spore germination, sporulation, and growth was determined. According to our results, *p*-hydroxybenzoic acid, ferulic acid, and cinnamic acid promoted spore germination, sporulation, and growth of FON. We also observed that *p*-hydroxybenzoic acid, ferulic acid, and cinnamic acid have a dose-dependent stimulatory effect. Ferulic acid and cinnamic acid have a greater stimulatory effect compared with *p-*hydroxybenzoic acid (**Figure 3**). At 40 μM and above, the germination, sporulation, and growth index were increased with increasing concentration of *p*-hydroxybenzoic acid, ferulic acid, and cinnamic acid. However, coumaric acid inhibited spore germination, sporulation, and growth of FON at a concentration above 20 μM, which showed that coumaric acid acts in a dose-dependent manner (**Figures 3A–C**).

### Effect of Wheat Intercropping on Phenolic Acid and Organic Acid Secretion of Watermelon Roots

**FIGURE 4:**
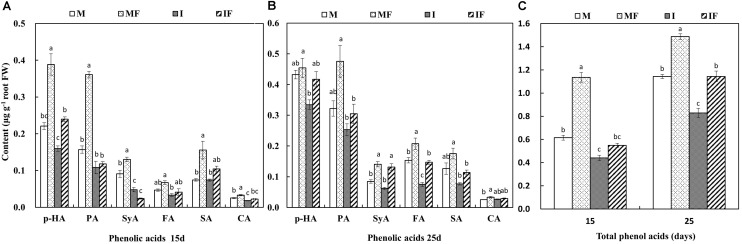
Quantity of phenolic acids in the root exudates of watermelon on day 15 **(A)** and day 25 **(B)**, and total phenolic acids **(C)** after FON inoculation. *P*-HA, *p*-hydroxybenzoic acid; PA, phthalic acid; SyA, syringic acid; FA, ferulic acid; SA, salicylic acid; CA, cinnamic acid. All values are presented as mean ± SE. The different letters on the mean values of the same phenolic acid and the same day indicate significant differences among the treatments (*P* < 0.05, Tukey’s test).

Most of the phenolic acids present in the root exudates of watermelon in the monocropping system were significantly increased after FON infection (**Figure 4**). Similarly, phenolic acids were also found to be higher in FON-inoculated plants on day 25 compared with day 15 (**Supplementary Figure S5**). However, the quantity of phenolic acids secreted from watermelon roots was less in the intercropping system compared with the monocropping system on days 15 and 25 after FON inoculation. The quantity of syringic acid was dramatically reduced in the root exudates of watermelon on day 15 after FON inoculation by wheat intercropping (**Figures 4A,B**).

**FIGURE 5:**
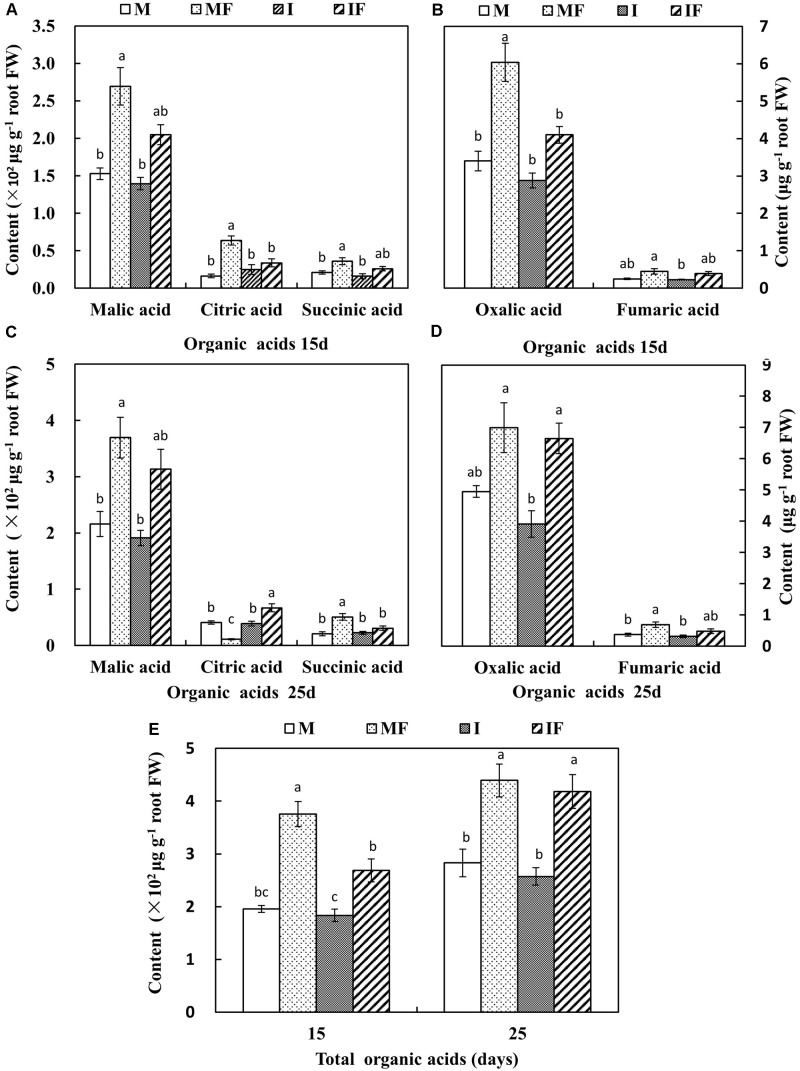
Quantity of organic acids in the root exudates of watermelon on day 15 **(A,B)** and day 25 **(C,D)**, and total organic acids **(E)** after FON inoculation. All values are presented as mean ± SE. The different letters on the mean values of the same organic acid and the same day indicate significant differences among the treatments (*P* < 0.05, Tukey’s test).

The secretion of organic acids from the roots of FON-inoculated watermelon plants was higher compared with non-inoculated watermelon plants except for citric acid secretion on day 25 after FON inoculation (**Figure 5**). The quantity of malic acid, citric acid, and oxalic acid secreted from the roots of watermelon was 24.0–46.9% higher in the monocropping system compared with the intercropping system on day 15 after FON inoculation (**Figures 5A,B**). The quantity of succinic acid was also significantly increased in the monocropping system compared with the intercropping system on day 25 after FON inoculation (**Figures 5C,D**).

### Effect of Wheat Intercropping on SA Biosynthesis of Watermelon

**FIGURE 6:**
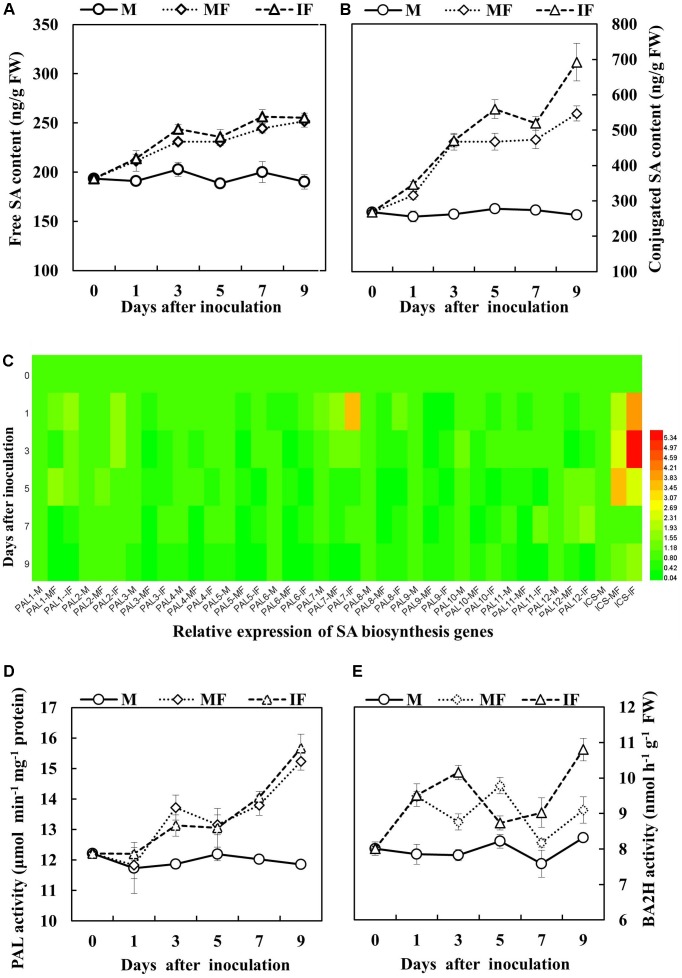
Salicylic acid (SA) accumulation in the roots of watermelon in response to FON inoculation. **(A)** Free SA content. **(B)** Conjugated SA content. **(C)** Expression analysis of PAL and ICS genes using matrix cluster analysis. The intensity of the red or green color represents the extent of up-regulation of the tested gene. **(D,E)** PAL and BA2H activities of watermelon roots in response to FON infection. All values are presented as mean ± SE.

We measured the free and conjugated SA content of watermelon after FON inoculation to determine the SA responses to FON infection. The level of SA remained constant until the 9th day in non-inoculated plants; however, both forms of SA (free and conjugated) were increased after 1 day of FON inoculation and remained higher for 9 days. Moreover, the quantity of free and conjugated SA in wheat-intercropped watermelon was higher compared with watermelon grown under the monocropping system (**Figures 6A,B**). Gene expression analysis showed that the transcript levels of genes in the *PAL* family were decreased after FON inoculation with the exception of *PAL1*, *PAL2*, *PAL7*, and *PAL12* (**Figure 6C**). The relative expression of *PAL1*, *PAL2*, and *PAL12* was slightly increased after FON inoculation; the relative expression of *PAL7* was obviously increased and this was highest on day 1 after FON inoculation. Interestingly, the expression of the *ICS* gene was apparently increased by up to 2.5- to 5.9-fold and 1.7- to 3.6-fold in the intercropping system and the monocropping system, respectively, from days 1 to 5 after FON inoculation (**Figure 6C**). Furthermore, enzyme activities of PAL and BA2H were significantly increased in the roots of watermelon grown under the intercropping system compared with the monocropping system after FON inoculation. The increased activities of these enzymes seem involved in SA biosynthesis through the PAL pathway (**Figures 6D,E**).

### Relative Expression of Defense-Related Genes

**FIGURE 7:**
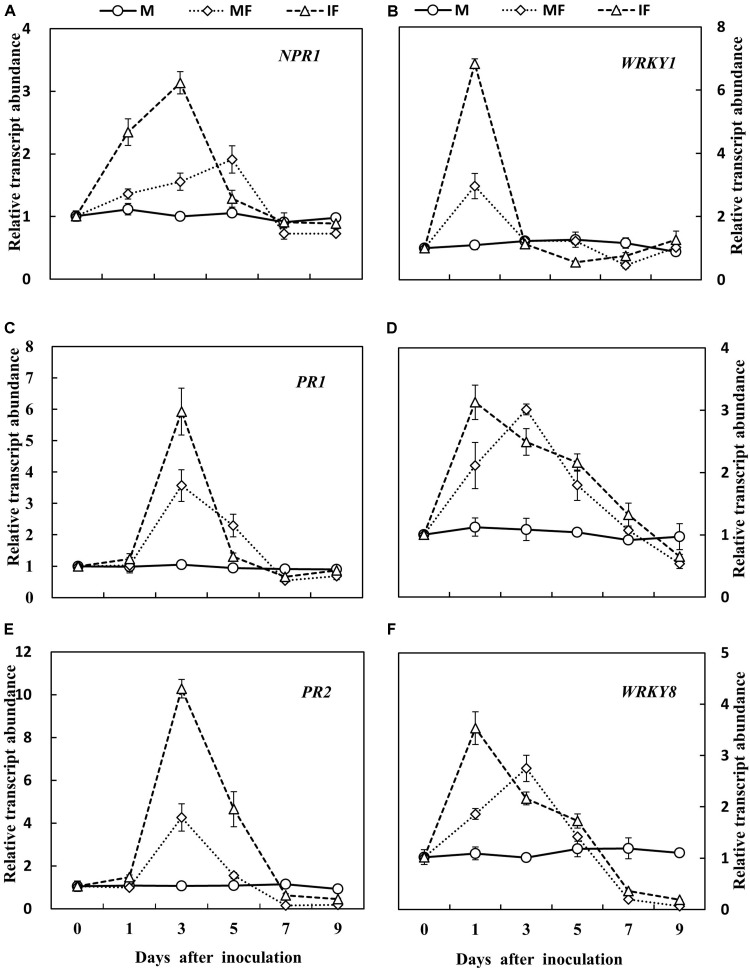
Gene expression analysis of six defense-related genes of watermelon roots in response to FON infection. **(A)** Expression pattern of *NPR1* gene in watermelon; **(C,E)** expression pattern of *PR* genes in watermelon; **(B,D,F)** expression pattern of *WRKY* genes in watermelon. All values are presented as mean ± SE.

**FIGURE 8:**
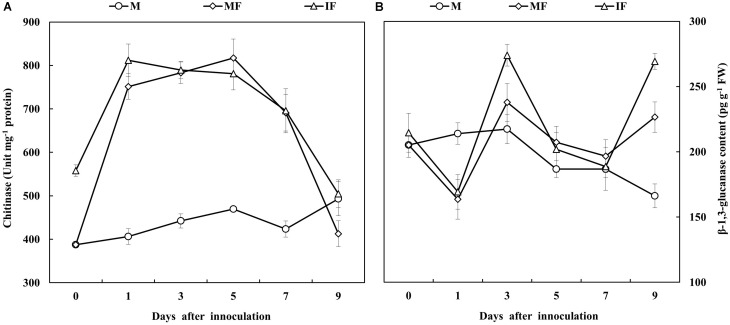
Chitinase **(A)** and β-1,3-glucanase **(B)** activities of watermelon roots in response to FON infection. All values are presented as mean ± SE.

Gene expression analysis showed that transcript abundance of *NPR1*, *PR1*, *PR2*, *WRKY1*, *WRKY6*, and *WRKY8* genes in the roots of watermelon was induced after FON inoculation. The relative expression of *NPR1* was apparently increased on day 1 after FON inoculation. This was highest on days 3 and 5 in the intercropping system and the monocropping system, respectively, after FON inoculation. The relative expression of *PR1* and *PR2* was increased, and the level of expression was highest on day 3 after FON inoculation in the intercropping system. The relative expression of *WRKY1* was highest on day 1 after FON inoculation in the intercropping system, and this was also higher compared with the monoculture system. Increased relative expression of *WRKY6* was observed on days 1, 3, 5, and 7, and the maximum level of relative expression was observed on day 1 after FON inoculation. Moreover, the relative expression of *WRKY6* was higher in the intercropping system compared with the monocropping system. Enhanced transcript abundance was observed for *WRKY8* in response to FON infection on day 1, and this was highest on days 3 and 1 in the monocropping system and the intercropping system, respectively (**Figure 7**). Furthermore, the activities of chitinase and β-1,3-glucanase were increased by 8.0 and 15.2% in the intercropping system compared with the monocropping system on days 1 and 3 after FON inoculation, respectively (**Figures 8A,B**).

## Discussion

*Fusarium* wilt of watermelon is among the important diseases of watermelon that cause significant crop losses (Zhou and Everts, 2004). This study showed that the incidence of *Fusarium* wilt of watermelon was reduced when it was intercropped with wheat that suppresses the FON population and growth (**Figures 1B,C**). The reduction of FON population in the intercropping system seems responsible for the low incidence of *Fusarium* wilt of watermelon (Ren et al., 2008; Xu et al., 2015). The results of this study indicated that the *Fusarium* wilt of watermelon under the intercropping system was substantially suppressed, likely because the growth and population of FON was reduced with the application of root exudates of wheat (**Figures 1C**, **2**).

We also observed that the growth of watermelon was improved in the wheat intercropping system compared with the monocropping system after FON inoculation (**Figure 1A**). A similar observation was also reported by Xiao et al. (2013); they observed that garlic intercropping significantly increased shoot and root biomass of cucumber. Wheat intercropping in watermelon promoted plant growth, probably because of reduced incidence of *Fusarium* wilt of watermelon.

Root exudates play a central role in regulating soil-borne pathogens. Roots secrete different compounds including sugars, phenolic acids, organic acids, amino acids, and other secondary metabolites that are involved in bacterial or fungal associations of pathogens in the rhizosphere (Brencic and Winans, 2005; Yao and Allen, 2006). Ferulic acid and cinnamic acid are considered as autotoxins for plants because they increase bacterial and fungal populations in the rhizosphere (Blum et al., 2000; Zhou and Wu, 2012b), enhance conidial germination of FON, and increase the incidence of *Fusarium* wilt (Ye et al., 2004, 2006). *p*-Hydroxybenzoic acid suppresses cucumber growth and increases the fungal communities and populations in the rhizosphere (Zhou et al., 2012). Our results indicated the severe incidence of *Fusarium* wilt of watermelon was observed in a monocropping system, which was likely caused by the root exudates of watermelon (**Figure 2**). The roots of watermelon secret phenolic acids such as *p*-hydroxybenzoic acid, ferulic acid, and cinnamic acid, and these acids stimulate spore germination, sporulation, and growth of FON (**Figure 3**). On the contrary, coumaric acid is a phenolic acid that plays an important role in protecting plants from environmental stresses, particularly pathogens (Hao et al., 2010; Lanoue et al., 2010; Zhou and Wu, 2012c). Interestingly, we found that the roots of wheat secrete coumaric acid that suppressed the spore germination, sporulation, and growth of FON (**Figure 3**), and this seems to be involved in the reduction of *Fusarium* wilt of watermelon.

Organic acids are also the main component of root exudates. Some organic acids stimulate biofilm formation of *B. amyloliquefaciens* SQR9 (Liu et al., 2014). According to the results of our study, five different organic acids were secreted from the roots of watermelon (**Supplementary Figure S4B**). However, to what extent these organic acids affect the growth of FON remains unclear. Thus, more information regarding these allelochemicals is required.

Chaparro et al. (2013) reported that root exudates are induced in the root zone by microorganism of rhizosphere; when roots are attacked by pathogens, the level of putative antimicrobial phenolic compounds is increased. Mostly these compounds contain nitrogen and carbon and serve as a source of nutrition for the microbes found in the rhizosphere. Fungal pathogens also depend on these exogenous carbon sources for their growth because they belong to heterotrophic organisms (Jones et al., 2004). In this study, sufficient quantities of phenolic acids and organic acids were secreted from the roots of FON-inoculated watermelon plants (**Figures 4**, **5**), and similar results had previously been observed when plants were infected with *Fusarium* (Lanoue et al., 2010; Ren et al., 2015). In the monocropping system, higher amounts of phenolic acids and organic acids were observed after FON inoculation, whereas the quantities of these compounds in the wheat intercropping system was at a normal level after some period of FON inoculation (**Figures 4C**, **5E**). Thus, wheat intercropping appears to protect the roots of watermelon from FON infection and limits the secretion of phenolic acids and organic acids, resulting in restricted growth of FON, which reduces the incidence of *Fusarium* wilt of watermelon.

Salicylic acid biosynthesis is induced after the infection of pathogens and under abiotic stresses such as ozone exposure and UV radiation. Altered levels of SA are extensively reported for a large number of mutants and transgenic plants under biological stress, particularly during infections (Vlot et al., 2009). In this study, we observed that free and conjugated SA persistently accumulated in the roots of watermelon after FON inoculation (**Figures 6A,B**). Meanwhile, expression of the *ICS* gene was significantly enhanced in watermelon roots after FON inoculation. Similarly, the relative gene expression of *PAL1*, *PAL2*, *PAL7*, and *PAL12* and the enzymatic activities of PAL and BA2H were also enhanced (**Figures 6C–E**), which indicates that SA had accumulated in the roots of watermelon through ICS and PAL pathways under FON-inoculated conditions. ICS is encoded by *ICS1* and *ICS2* in *Arabidopsis*, *ICS1* plays a primary role in SA biosynthesis in response to plant–pathogen interactions (Wildermuth et al., 2001). Interestingly, the concentration of SA was higher in watermelon grown in the wheat intercropping system compared with the monocropping system (**Figures 6A,B**).

Salicylic acid is involved in the regulation of PR protein expression leading to the initiation of plant defense-responses against biotrophic pathogens (Dempsey et al., 2011). The up-regulation of defense-related genes such as *PR1* and *PR2* was associated with lesion limitation in the roots of soybean seedlings inoculated with *Phytophthora sojae* and imparted a partial resistance against the disease (Upchurch and Ramirez, 2010). The overexpression of *NPR1* in *Arabidopsis* enhanced the resistance against *F. graminearum* (Makandar et al., 2010). According to another report, *WRKY6* and *WRKY8* genes were highly expressed in response to pathogen infection, but these genes were not expressed in SA-treated plants. However, the relative expression of *WRKY1* showed very little or no change after pathogen infection or SA treatment (Dong et al., 2003). In this study, It was also observed that the relative expression of *NPR1*, *PR1*, *PR2*, *WRKY1*, *WRKY6*, and *WRKY8* was increased in the wheat intercropping system, and the activities of chitinase and β-1,3-glucanase were also increased in the roots of watermelon after FON inoculation (**Figures 7**, **8A,B**). Intercropped plants produce allelopathic compounds that promote specific plant defense reactions in neighboring plants leading to protection against phytopathogens (Zhu et al., 2000; Bais et al., 2006). Chitinases and glucanases are distinct PR proteins that hamper pathogen growth (Durrant and Dong, 2004). The results of this study indicated that resistance of watermelon to FON was increased in the wheat intercropping system because of enhanced relative expression of disease-related genes and increased activities of PR enzymes. SA plays a signaling role in the activation of defense responses against microbial pathogens (Garcion and Métraux, 2006). *NPR1* acts as a positive regulator of the SA-mediated defense signaling pathway. *AtNPR1* expression promoted SA accumulation and *PR1* expression in *F. graminearum*-infected wheat and reduced the severity of *Fusarium* head blight (Makandar et al., 2012). The findings of this study indicate that *NPR1* expression in watermelon was associated with a systemic increase in SA accumulation and relative expression of *PR1*, *PR2*, *WRKY1*, *WRKY6*, and *WRKY8* in response to FON infection. Our results indicated that wheat intercropping increases the accumulation of SA in watermelon, and SA induces the defense-response of watermelon against FON infection. Through this mechanism, SA enhances watermelon resistance to FON.

## Conclusion

Wheat intercropping in watermelon reduces the population, germination, sporulation, and growth of FON, and suppresses the incidence of *Fusarium* wilt of watermelon, and contributes to improved plant growth and development. Coumaric acid is secreted from the roots of wheat, which inhibits spore germination, sporulation, and growth of FON. Moreover, wheat intercropping reduces the secretion of organic acids and phenolic acids from the roots of watermelon, leading to reduced availability of carbon- and nitrogen-containing compounds required for FON growth and colonization. The expression of disease-related defense-responsive genes and PR enzymes activities were higher in watermelon grown under the wheat intercropping system compared with the watermelon grown under the monocropping system. Considering the results of this study, it can be concluded that wheat intercropping in watermelon improves the resistance of watermelon to FON. Thus, wheat intercropping can be utilized as an effective strategy for the management of *Fusarium* wilt of watermelon.

## Author Contributions

HL and ZB conceived and designed the experiments. HL and HC performed the experiments and analyzed the data. HL and MN wrote the manuscript. HL, HC, MN, YH, QK, FC, HS, and ZB revised and finally approved this article for publication.

## Conflict of Interest Statement

The authors declare that the research was conducted in the absence of any commercial or financial relationships that could be construed as a potential conflict of interest.

## Supplementary Material

FIGURE S1 The watermelon plants on day 15 after FON inoculation. M, watermelon monocropping; MF, watermelon monocropping with FON inoculation; I, watermelon/wheat intercropping; IF, watermelon/wheat intercropping with FON inoculation.

FIGURE S2 The incidence of *Fusarium* wilt of watermelon on day 25 after FON inoculation. M, watermelon monocropping; MF, watermelon monocropping with FON inoculation; I, watermelon/wheat intercropping; IF, watermelon/wheat intercropping with FON inoculation.

FIGURE S3 HPLC chromatogram of the phenolic acids used for standard **(A)** root exudates from watermelon **(B)** and wheat **(C)**. The peaks from left to right in the standards represent the following standard compounds: 1, coumaric acid; 2, *p*-hydroxybenzoic acid; 3, phthalic acid; 4, syringic acid; 5, ferulic acid; 6, salicylic acid; 7, cinnamic acid.

FIGURE S4 HPLC chromatogram of the organic acids used for standard **(A)** and root exudates from watermelon **(B)** and wheat **(C)**. The peaks from left to right in the standards represent the following standard compounds: 1, oxalic acid; 2, malic acid; 3, citric acid; 4, succinic acid; 5, fumaric acid.

FIGURE S5 Quantity of phenolic acids in watermelon root exudates in the monocropping system **(A)** and in the intercropping system **(B)** on day 15 and day 25 after FON inoculation. *P*-HA, *p*-hydroxybenzoic acid; PA, phthalic acid; SyA, syringic acid; FA, ferulic acid; SA, salicylic acid; CA, cinnamic acid. All values are presented as mean ± SE. The different letters on the mean values of the same phenolic acid indicate significant differences between the treatments (*P* < 0.05, independent sample *t*-test).

TABLE S1 The list of primer sequences used for qRT-PCR analysis.
