# Supplementary figures and images for: Wheat Intercropping Enhances the Resistance of Watermelon to Fusarium Wilt
Source: Front Plant Sci. 2018 May 25;9:696. doi: 10.3389/fpls.2018.00696 (PMC5980984; doi:10.3389/fpls.2018.00696)

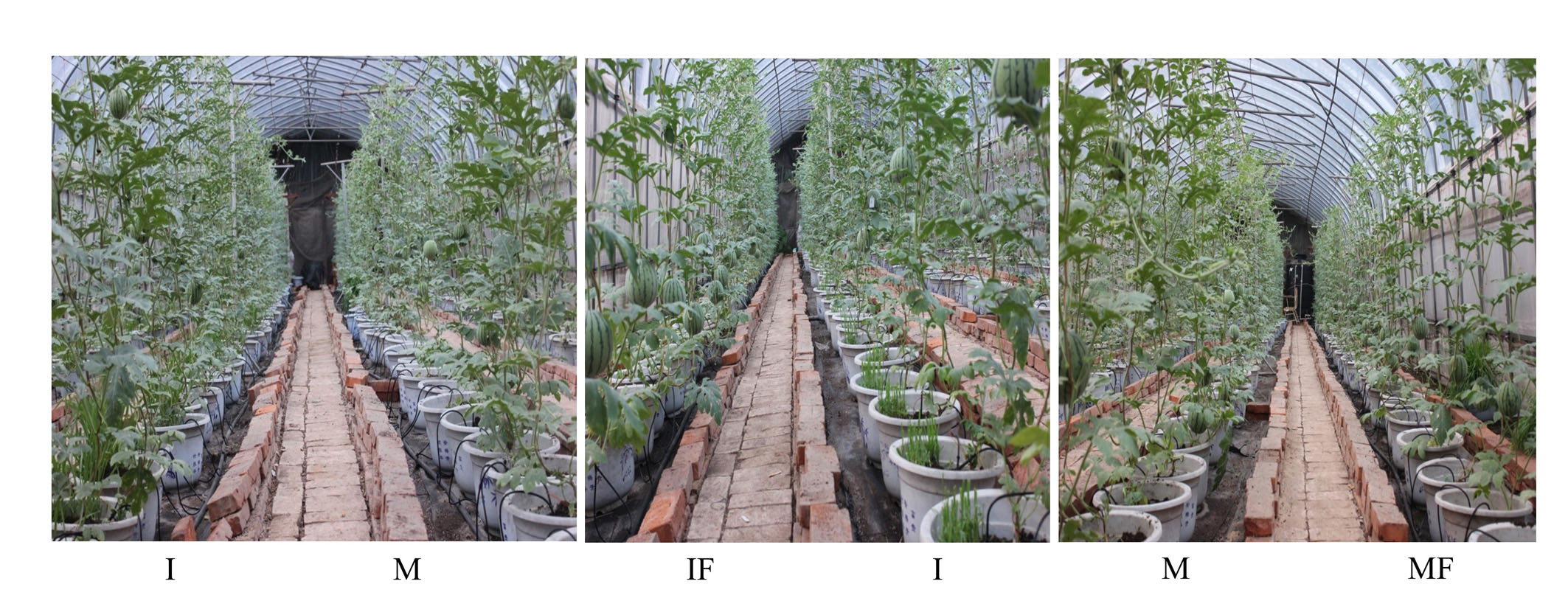

Supplement: FIGURE S1 — The watermelon plants on day 15 after FON inoculation. M, watermelon monocropping; MF, watermelon monocropping with FON inoculation; I, watermelon/wheat intercropping; IF, watermelon/wheat intercropping with FON inoculation. [file Image_1.JPEG]

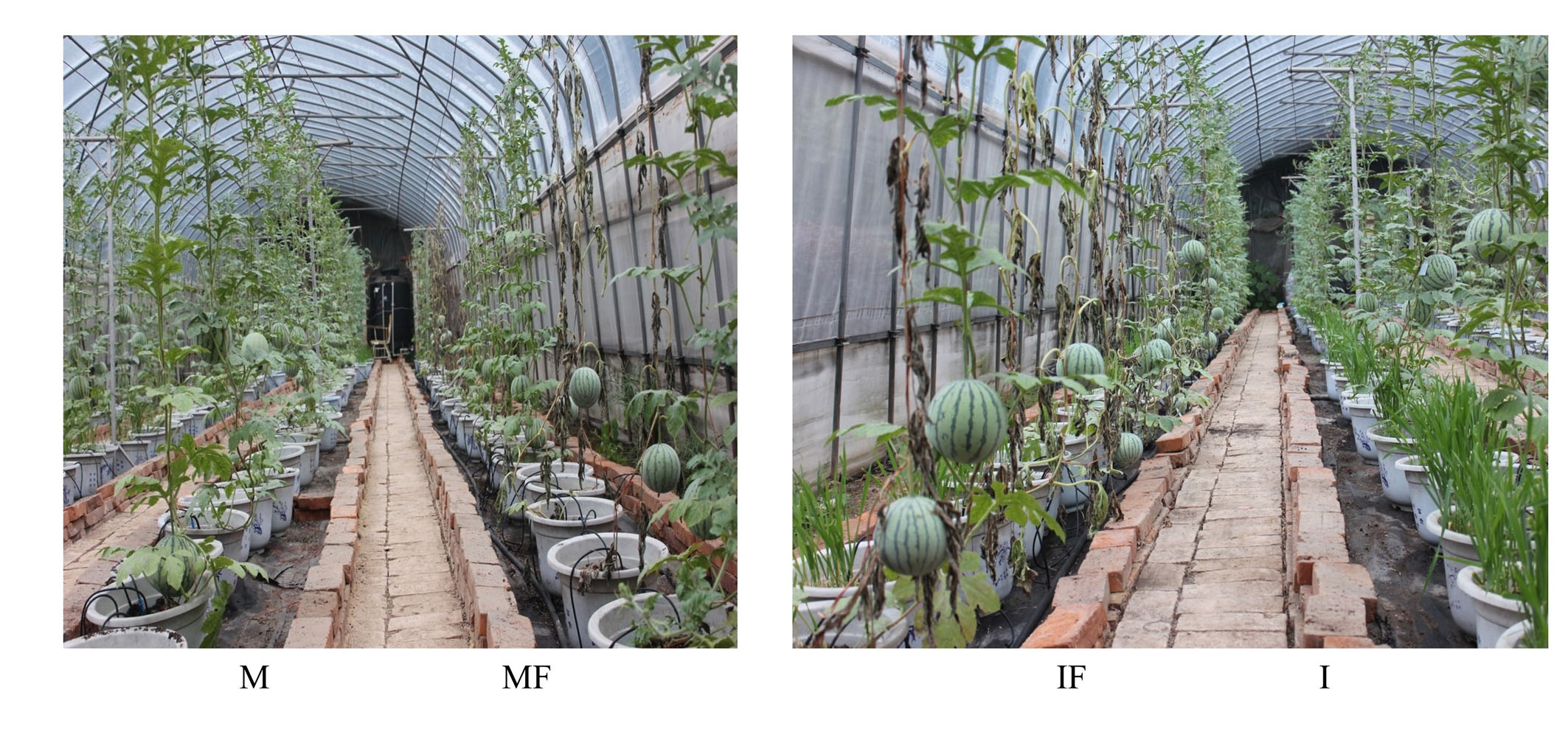

Supplement: FIGURE S2 — The incidence of Fusarium wilt of watermelon on day 25 after FON inoculation. M, watermelon monocropping; MF, watermelon monocropping with FON inoculation; I, watermelon/wheat intercropping; IF, watermelon/wheat intercropping with FON inoculation. [file Image_2.JPEG]

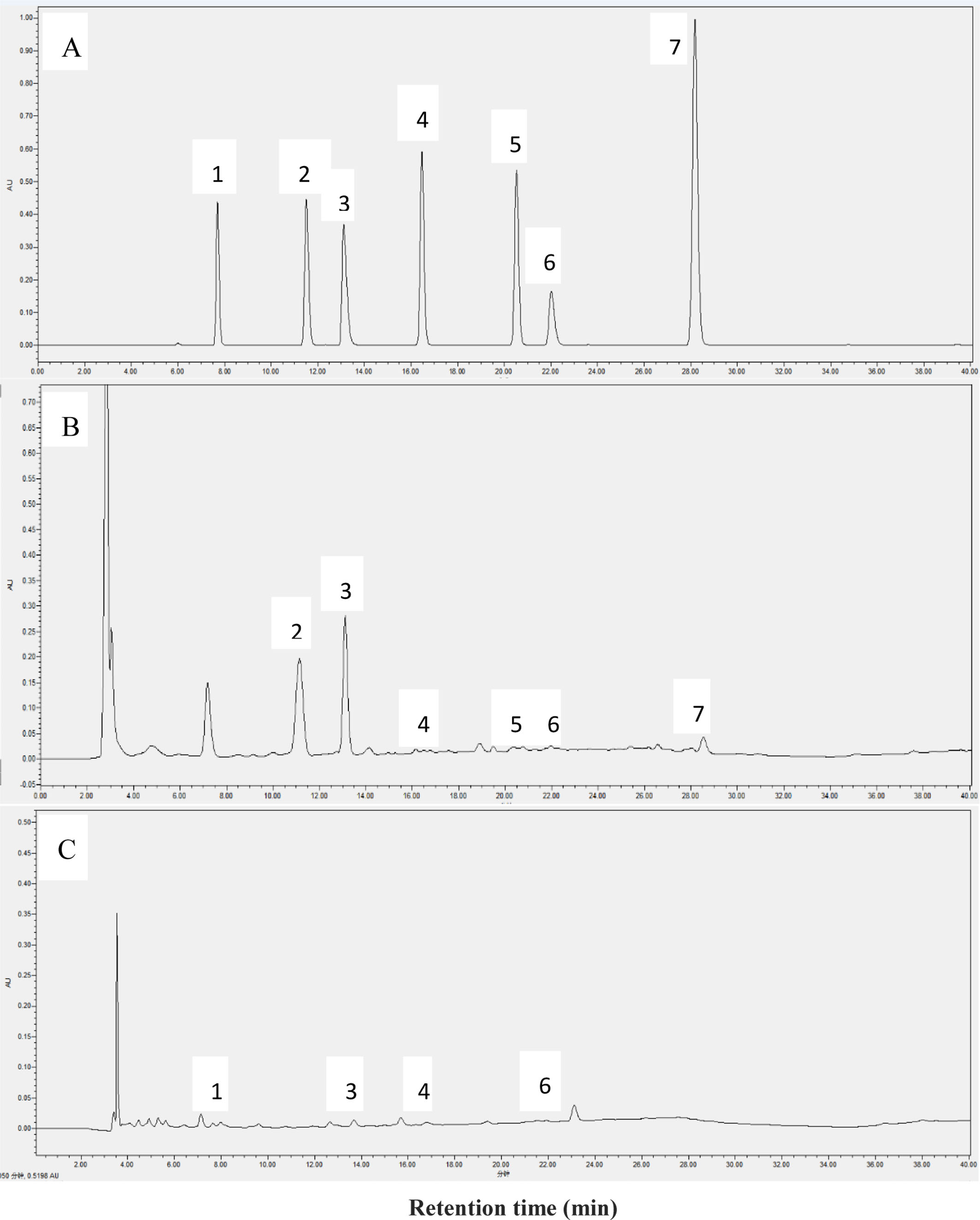

Supplement: FIGURE S3 — HPLC chromatogram of the phenolic acids used for standard (A) root exudates from watermelon (B) and wheat (C). The peaks from left to right in the standards represent the following standard compounds: 1, coumaric acid; 2, p-hydroxybenzoic acid; 3, phthalic acid; 4, syringic acid; 5, ferulic acid; 6, salicylic acid; 7, cinnamic acid. [file Image_3.JPEG]

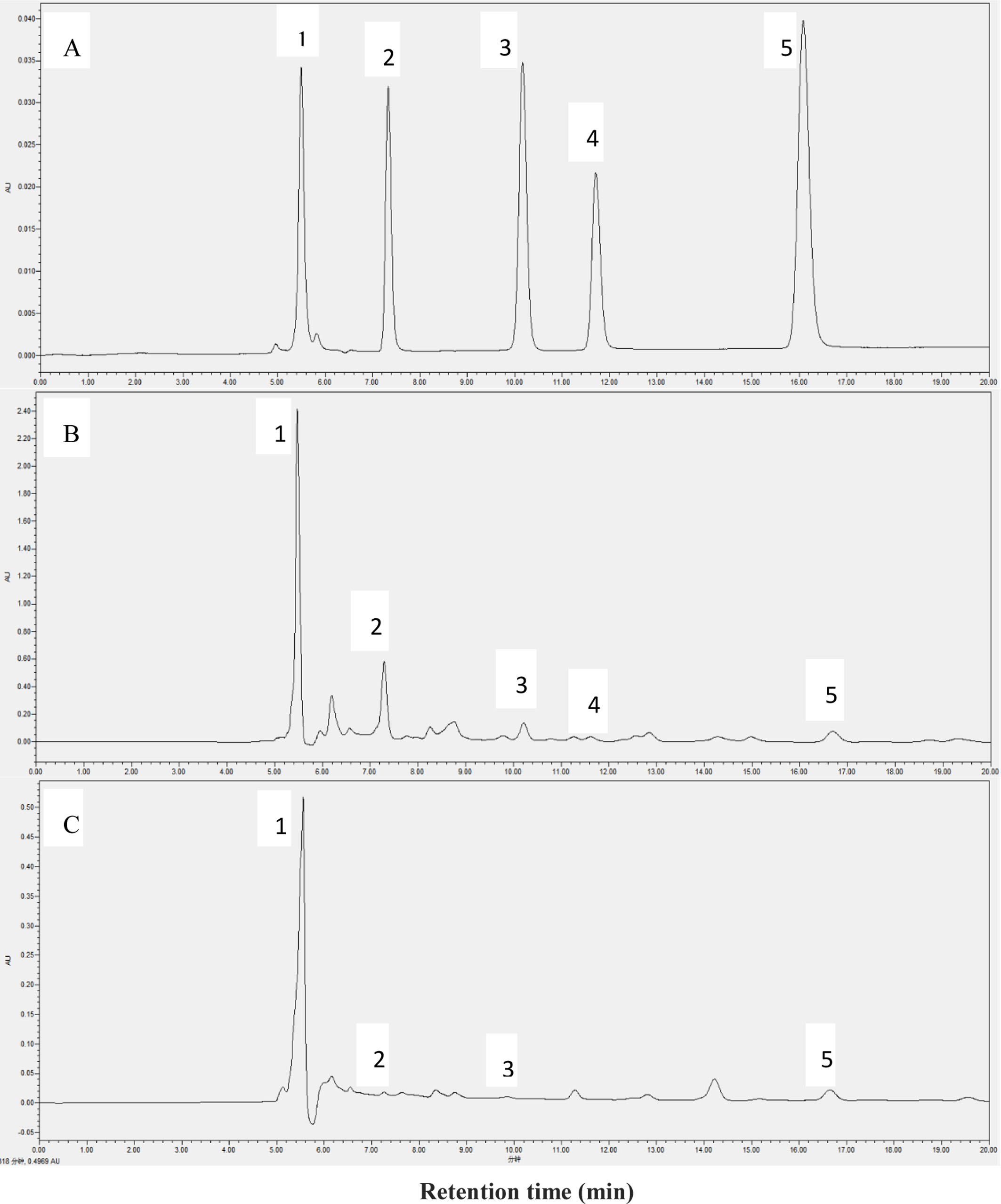

Supplement: FIGURE S4 — HPLC chromatogram of the organic acids used for standard (A) and root exudates from watermelon (B) and wheat (C). The peaks from left to right in the standards represent the following standard compounds: 1, oxalic acid; 2, malic acid; 3, citric acid; 4, succinic acid; 5, fumaric acid. [file Image_4.JPEG]

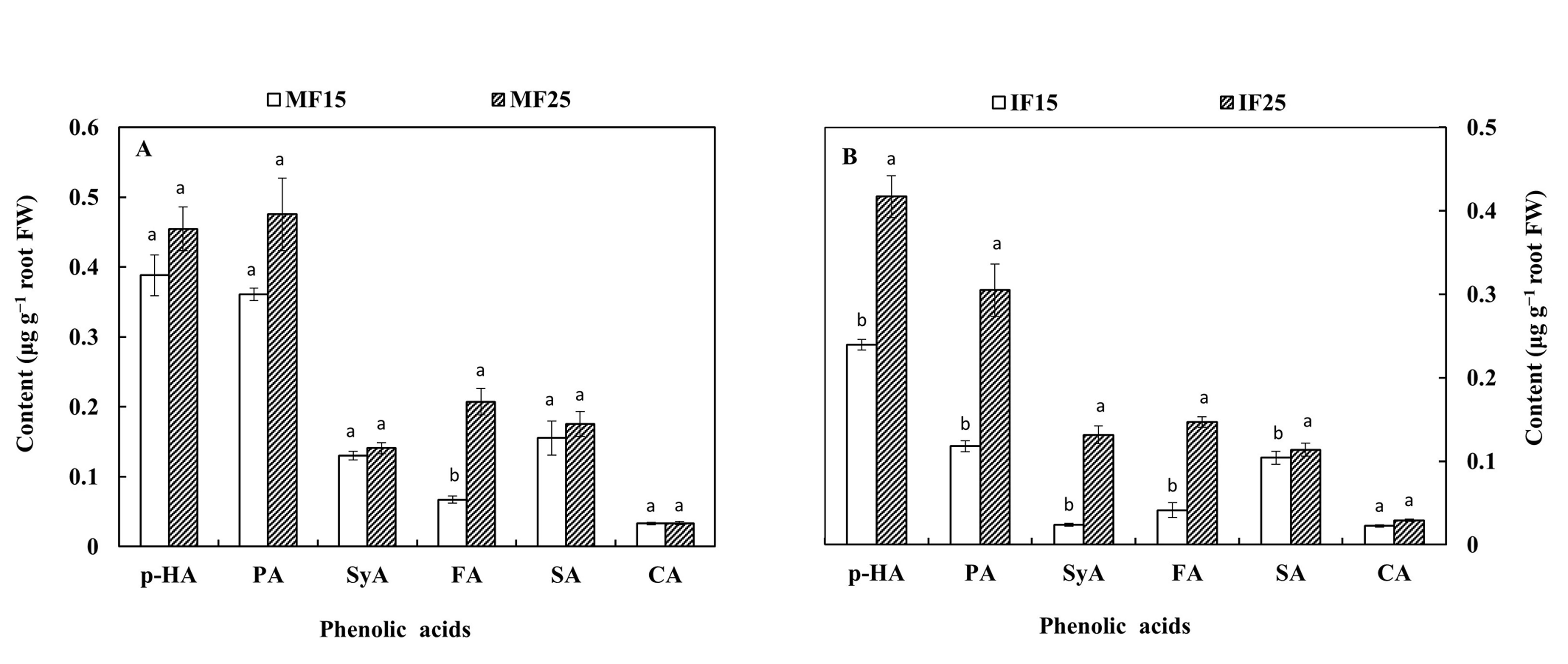

Supplement: FIGURE S5 — Quantity of phenolic acids in watermelon root exudates in the monocropping system (A) and in the intercropping system (B) on day 15 and day 25 after FON inoculation. P-HA, p-hydroxybenzoic acid; PA, phthalic acid; SyA, syringic acid; FA, ferulic acid; SA, salicylic acid; CA, cinnamic acid. All values are presented as mean ± SE. The different letters on the mean values of the same phenolic acid indicate significant differences between the treatments (P < 0.05, independent sample t-test). [file Image_5.JPEG]
