# Supplementary material for: Wheat Intercropping Enhances the Resistance of Watermelon to Fusarium Wilt
Source: Front Plant Sci. 2018 May 25;9:696. doi: 10.3389/fpls.2018.00696 (PMC5980984; doi:10.3389/fpls.2018.00696)
Supplement: TABLE S1 — The list of primer sequences used for qRT-PCR analysis. [file Table_1.docx]

**Supplementary Table 1** The list of primer sequences used for qRT-PCR analysis.

| Gene name | Gene | Forward primer 5'-3’ | Reverse primer 5'-3’ |
| --- | --- | --- | --- |
| *ClCAC* | *Cla020794* | AATTGTGGTTGATGCTGCAC | TGACAGCTGTACCTGGCATC |
| *ClTUA* | *Cla003129* | CTTGCTGGGAGCTCTATTGC | AACGGATTAAAAGCGTCGTG |
| *PAL1* | *Cla008727* | ATGAAACCCAACACTTGCAAT | ACTCCCCCACCATTTTCTT |
| *PAL2* | *Cla 011180* | AAGGCTGCCAACGCTCTCAG | ATCGCTTCGACGAGCAACGG |
| *PAL3* | *Cla018297* | GCCGGAACAATATCAACCATG | CATCCGCTTCACTTCTTCC |
| *PAL4* | *Cla018298* | TGCGCCATTACTACTCATCC | GCGCTTCACCTCATGAG |
| *PAL5* | *Cla018299* | TGACTTGAGGCACCTAGAAG | ATCTTGAAGGATGAAGTGTGC |
| *PAL6* | *Cla018300* | TGGCACCAACTCAATTCCAG | CCTCATCGAGATGGCTTCC |
| *PAL7* | *Cla018301* | TGCCCAATTGGAACAATGTG | TATACTCTTCCACCATCCTCTT |
| *PAL8* | *Cla018302* | TGGAGACCATCTGCAAGAG | GCGCTTCACCTCATCAAG |
| *PAL9* | *Cla018303* | CCCTAGTCAAGACCATTTGCAA | GCTTGACAAGAGGCCTCCGA |
| *PAL10* | *Cla012779* | ATGGCCCAAAAGGTTTGTGCT | ATACTCTTCCACCATCCGC |
| *PAL11* | *Cla012780* | GGCTTCCAAAAATA ATGATTC | TCCTCCAAGCTTCACCAAA |
| *PAL12* | *Cla013761* | ATCCGGAAAATGGCAACCAT | CCTCCAAGTCGCACAATAG |
| *ICS* | *Cla019128* | ACGGAGAGTCTGAGGAGAG | GCACTTGAAGCCGAATAATC |
| *NPR1* | *Cla009186* | 5'-TGCAATGCGTAAGGACCCAT-3' | 5'-AGTCCGACCATCCAGAGTCA-3' |
| *PR1* | *Cla001623* | 5'-ATGCCAACCAACGCATCAAC-3' | 5'-TGGCCAACCCCATGCAATAT-3' |
| *PR2* | *Cla009588* | 5'-CCGTCAACTGTGGTCACTGA-3' | 5'-CTCCGCCAACCTTCTCCAAT-3' |
| *WRKY1* | *Cla008104* | 5'-CCAGAGGCTAAGAGATGGCG-3' | 5'-GTGGTTTGGACGACGACTCT-3' |
| *WRKY6* | *Cla014433* | 5'-TTCCGAACCCGCTCATGAAA-3' | 5'-AGTTCATCGCTTTCACCCGT-3' |
| *WRKY8* | *Cla009748* | 5'-GGTGGCGCATCCGTTTATTC-3' | 5'-TGCCGGTTGAAGAACGATGA-3' |
| *FON* |  | 5'-CGATTAGCGAAGACATTCACAAGACT-3' | 5'-ACGGTCAAGAAGATGCAGGGTAAAGGT-3' |

Note: The watermelon *ClCAC* and *ClTUA* genes were used as reference genes.
